# A Temperature-Compensation Technique for Improving Resolver Accuracy

**DOI:** 10.3390/s21186069

**Published:** 2021-09-10

**Authors:** Wandee Petchmaneelumka, Vanchai Riewruja, Kanoknuch Songsuwankit, Apinai Rerkratn

**Affiliations:** School of Engineering, King Mongkut’s Institute of Technology Ladkrabang, Ladkrabang, Bangkok 10520, Thailand; wandee.pe@kmitl.ac.th (W.P.); kanoknuch.so@kmitl.ac.th (K.S.); apinai.re@kmitl.ac.th (A.R.)

**Keywords:** resolver, inductive transducer, temperature-compensation technique, subtract-and-sum circuit, opamp

## Abstract

Variation in the ambient temperature deteriorates the accuracy of a resolver. In this paper, a temperature-compensation technique is introduced to improve resolver accuracy. The ambient temperature causes deviations in the resolver signal; therefore, the disturbed signal is investigated through the change in current in the primary winding of the resolver. For the proposed technique, the primary winding of the resolver is driven by a class-AB output stage of an operational amplifier (opamp), where the primary winding current forms part of the supply current of the opamp. The opamp supply-current sensing technique is used to extract the primary winding current. The error of the resolver signal due to temperature variations is directly evaluated from the supply current of the opamp. Therefore, the proposed technique does not require a temperature-sensitive device. Using the proposed technique, the error of the resolver signal when the ambient temperature increases to 70 °C can be minimized from 1.463% without temperature compensation to 0.017% with temperature compensation. The performance of the proposed technique is discussed in detail and is confirmed by experimental implementation using commercial devices. The results show that the proposed circuit can compensate for wide variations in ambient temperature.

## 1. Introduction

A resolver, which is a kind of inductive transducer, is a useful device in instrumentation and measurement systems. The operation of a resolver is identical to a variable transformer, which consists of a primary winding as a rotor and two secondary windings placed at right angles from each other as stators [1,2,3,4]. Resolvers are widely used for the measurements of the angle, position, and speed in instrumentation and control systems. Resolvers provide high reliability, durability, and resolution. Therefore, resolvers are especially suitable for harsh environments. Resolvers have been applied in industries, military equipment, robots, aerospace, radars, electric vehicles, and medical and scientific equipment [5,6,7]. Practically, the primary winding of a resolver is excited by a sinusoidal signal. The resolver signals from the two secondary windings known as the quadrature signals are proportional to the sine and cosine functions of the rotor shaft angle, which are in the form of amplitude modulation with a suppressed carrier. Traditionally, a synchronous demodulator based on an analog multiplier, low-pass filter, or analog switch and integrator has been used to extract the shaft-angle signals from the resolver signals [1,8,9,10]. However, the response time and accuracy of these demodulators deteriorate due to the large time constant and phase shift caused by the dominant pole of the low-pass filter and integrator. To overcome these disadvantages, an alternative technique using a peak-amplitude finder was introduced [3,11,12]. This technique provides a simple configuration and rapid response. Recently, techniques for determining position from a resolver signal in digital form have been proposed in the literature [5,13,14,15,16,17]. Unfortunately, these approaches require the resolver to operate at a constant ambient temperature to achieve this accurately.

Many parameters can affect the accuracy of the resolver, such as phase shifts between the primary winding and secondary windings, amplitude imbalance and imperfect quadrature between two resolver signals, and variation in the ambient temperature [1,3,18]. Amplitude imbalance and imperfect quadrature cause errors in the determination of the shaft angle. Recently, a technique to minimize the error of the shaft-angle determination caused by amplitude imbalance and imperfect quadrature was proposed [18]. However, this technique requires a high-speed processor. Consequently, a complex measurement system structure is required. An amplitude imbalance or phase shift between the primary and secondary winding can be prevented using a technique proposed in the literature [3]. Variation in the ambient temperature influences the resistance of the winding, the mutual inductance, the magnetizing current, and the core loss current of the resolver, causing amplitude error in the resolver signals. The traditional technique to minimize this temperature effect is based on the use of an inverse tangent of the ratio of two resolver signals [1,4,8]. However, the disadvantage of this technique is that a large error occurs when the resolver signal is close or equal to zero. The response time is also rather slow for this technique. An approach based on the use of a temperature-sensitive device was developed to compensate for the temperature effect of inductive transducers [19,20,21,22]. The temperature effect in the technique mentioned above is compensated for in an open-loop procedure, which causes inaccuracies in the resolver signal. Other techniques are based on the modification of the transducer’s structure or material to obtain low-temperature sensitivity [23,24,25], and the use of reference inductance to compensate for the temperature effect [26]. The authors of [27] constructed a closed-loop technique to compensate for the temperature of the inductive transducer without using a temperature sensor. This technique requires the output signals of the transducer to be in linear form, which is only suitable for transducers used for the measurement of linear displacement, such as linear variable differential transformers (LVDTs). In contrast, the resolver signals are in the sine and cosine functions. Therefore, the techniques mentioned above are not applicable to these resolvers.

The purpose of this study was to construct a temperature-compensation technique for a resolver without requiring a temperature-sensitive device. From the transformer principle, the parameters of the secondary winding side can be referred to the primary winding side [28]. Therefore, the error of the resolver signal on the secondary winding side because of temperature can be extracted from the primary winding side. In this study, the error in the resolver signal due to the temperature effect was investigated from the current flowing through the primary winding of the resolver using the opamp supply-current-sensing technique [29,30,31]. The amplitude variation in the resolver signal due to the temperature effect was determined and compensated for using subtract-and-sum topology. The performance of the proposed technique was analyzed and confirmed by experimental implementation. All the devices used in the proposed technique are commercially available. Hence, the attractions of the proposed technique are its performance, simple configuration, and low cost.

## 2. Principle

**Figure 1:**
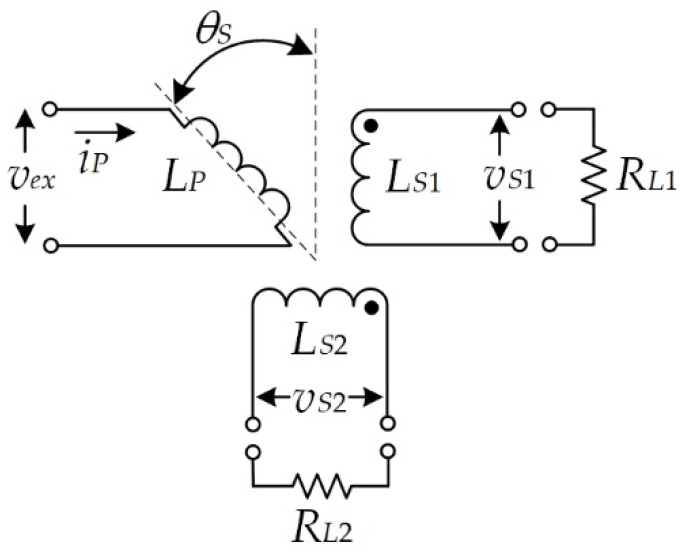
Principle of the functioning of the resolver.

The principle of the resolver is shown in Figure 1 [1]. A sinusoidal signal *v_ex_* = *V_P_* sin (*ω_ex_t*) is applied to the primary winding *L_P_*, where *V_P_* is the peak amplitude voltage, *ω_ex_* = 2*πf_ex_* is the angular frequency, and *f_ex_* is the frequency of the excitation signal. Two secondary windings are terminated by the load resistors *R_L_*_1_ = *R_L_*_2_, and the resolver signals *v_S_*_1_ and *v_S_*_2_ are produced in form of quadrature sinusoidal signals as

(1) $$v_{S1}=k_{RS}v_{ex}\sin(\theta_{S})=k_{RS}V_{P}\sin(\omega_{ex}t)\sin(\theta_{S})$$

(2) $$v_{S2}=k_{RS}v_{ex}\cos(\theta_{S})=k_{RS}V_{P}\sin(\omega_{ex}t)\cos(\theta_{S})$$

where *k_RS_* denotes the transformation ratio of the resolver and *θ_S_* is the shaft angle. Practically, the amplitude of the resolver signals is affected by variations in the ambient temperature because the mutual inductance, the resistance of both the primary and secondary windings, the magnetizing current, and the core loss current of the resolver depend on variations in the ambient temperature.

Therefore, the transformation ratio *k_RS_* is affected by the ambient temperature. The resolver signals from Equations (1) and (2) can be written as

(3) $$v_{S1}=k_{RS}(1-\alpha \Delta T)V_{P}\sin(\omega_{ex}t)\sin(\theta_{S})$$

(4) $$v_{S2}=k_{RS}(1-\alpha \Delta T)V_{P}\sin(\omega_{ex}t)\cos(\theta_{S})$$

where α and Δ*T* are the temperature coefficient of the resolver and the temperature deviation from room temperature at 25 °C, respectively. From Equations (3) and (4), sin (*ω_ex_t*) can be simply removed by a synchronous demodulator. Thus, the resolver signals can be expressed as

(5) $$v_{S1}=k_{RS}(1-\alpha \Delta T)V_{P}\sin(\theta_{S})$$

(6) $$v_{S2}=k_{RS}(1-\alpha \Delta T)V_{P}\cos(\theta_{S})$$

**Figure 2:**
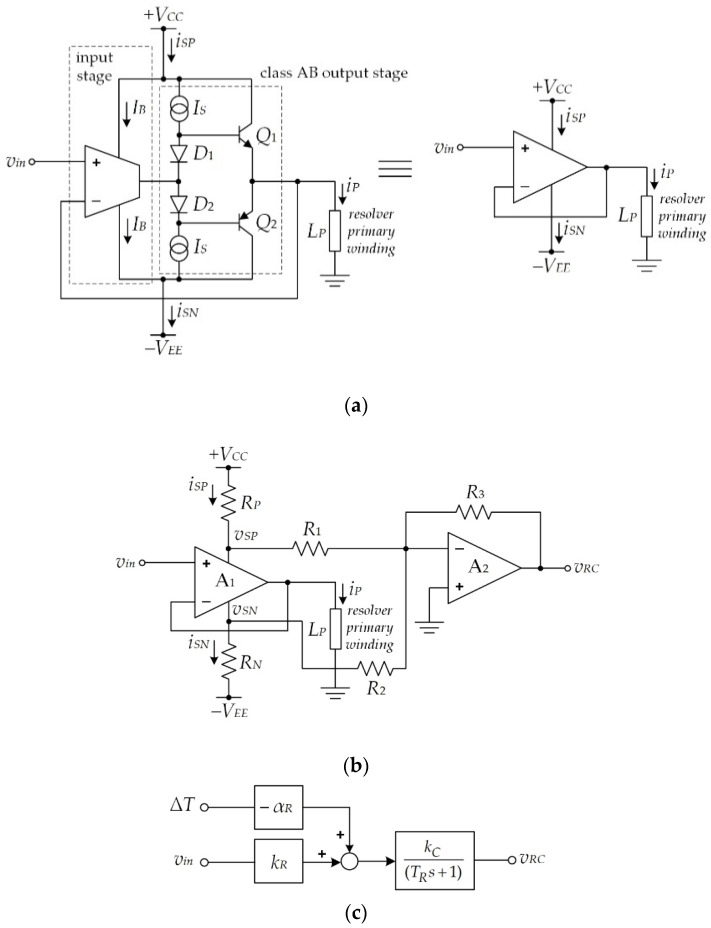
(**a**) Opamp equivalent circuit; (**b**) opamp supply-current sensing technique; (**c**) block diagram of primary winding current extraction including the temperature effect.

The amplitude of the resolver signals depends on variation in the ambient temperature. The variation in the ambient temperature affects all the quantities of the resolver and causes deviation in the transformation ratio *k_RS_*. Based on the principle on which transformers function, the secondary winding currents flowing through the load resistors *R_L_*_1_ and *R_L_*_2_ can be referred to the primary side [28]. Therefore, the primary winding current *i_P_* can be used to investigate the secondary winding current. The disturbance of the primary winding current *i_P_* (which includes the magnetizing current and the core loss current) by the ambient temperature can be approximated as

(7) $$i_{P}=\frac{\left(1-\alpha \Delta T\right)v_{ex}}{\left|Z_{PL}\right|}=(1-\alpha \Delta T)k_{R}v_{ex}$$

where *k_R_* = 1/|*Z_PL_*|, and *Z_PL_* is the combination of the primary-side impedance and the secondary-side impedance referred to the primary side of the resolver. It should be noted that the changes in the magnetizing current and the core loss current due to temperature are very small compared to the change in the secondary winding current, which can be considered as negligible [25,32]. If the temperature effect on the primary winding current is compensated for, then the secondary winding current is compensated simultaneously. Practically, the primary winding *L_P_* of the resolver is driven by the opamp, as shown in Figure 2a. From Figure 2a, the output state of the opamp is generally a class-AB configuration where the output current of the opamp exists within its supply current. In this study, a bipolar opamp was used for experimental implementation. From Figure 2a, the supply currents *i_SP_* and *i_SN_* of the opamp can be expressed as [29,30,31]

(8) $$i_{SP}=I_{B}+\frac{\sqrt{\left(i_{P}^{2}+4I_{S}^{2}\right)}+i_{P}}{2}\;\;for\;\left|i_{P}\right|<2I_{S}$$

and

(9) $$i_{SN}=I_{B}+\frac{\sqrt{\left(i_{P}^{2}+4I_{S}^{2}\right)}-i_{P}}{2}\;\;for\;\left|i_{P}\right|<2I_{S}$$

where *I_B_* and *I_S_* denote the quiescent current and the bias current of the class-AB output state of the opamp, respectively.

For the large-amplitude primary winding current *i_P_* or |*i_P_*| > 2*I_S_*, the currents *i_SP_* and *i_SN_* can be described by

(10) $$i_{SP}=\left\{ \begin{array}{l} I_{B}+i_{P}\;\;\;for\;i_{P}>2I_{S}\\ I_{B}\;\;\;\;\;\;for\;i_{P}<-2I_{S} \end{array}\right.$$

and

(11) $$i_{SN}=\left\{ \begin{array}{l} I_{B}\;\;\;\;for\;i_{P}>2I_{S}\\ I_{B}+i_{P}\;\;\;for\;i_{P}<-2I_{S} \end{array}\right.$$

The primary winding current *i_P_* of a resolver affected by temperature can be observed from the supply current of the opamp. The schematic diagram used to achieve the supply current of the opamp is shown in Figure 2b. From the circuit in Figure 2b, if the input signal *v_ex_* is applied, then the primary winding current *i_P_* can be described by *v_ex_*/*Z_PL_*. The resistors *R_P_* and *R_N_* convert the current signals *i_SP_* and *i_SN_* to the voltage signals *v_SP_* and *v_SN_*, respectively. Opamp *A*_2_ and the resistors *R*_1_, *R*_2_, and *R*_3_ function as a summing amplifier to sum the voltage signals *v_SP_* and *v_SN_* [30,31]. Thus, the primary winding current *i_P_* of the resolver is determined in the form of the voltage signal *v_RC_*. The voltage signal *v_RC_* can be stated as

(12) $$v_{RC}=\frac{R_{P}R_{3}}{R_{1}}i_{SP}+\frac{R_{N}R_{3}}{R_{2}}i_{SN}$$

Practically, the resistances *R*_1_ = *R*_2_ and *R_P_* = *R_N_* are assigned. If the peak amplitude of the current signal *i_P_* is greater than 2*I_S_* or |*i_P_*| > 2*I_S_*, then Equation (12) can be written as

(13) $$v_{RC}=\frac{R_{P}R_{3}}{R_{1}}i_{P}=(1-\alpha \Delta T)k_{C}k_{R}v_{in}$$

**Figure 3:**
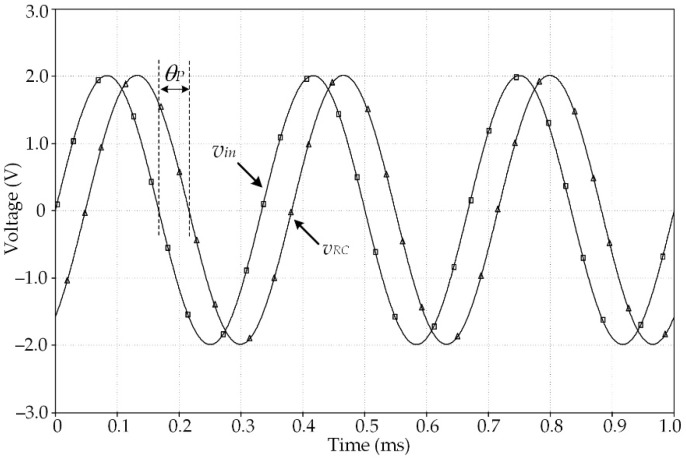
Simulation result of Figure 2b.

The primary winding current of the resolver can be determined from the voltage signal *v_RC_*. Practically, the parameter *k_C_* is normally assigned as 1/*k_R_*. The simulation result of the circuit depicted in Figure 2b obtained using the PSPICE analog simulation program is shown in Figure 3, where a sinusoidal signal of 3 kHz with a peak amplitude of 2V is applied as the input signal *v_in_*. The parameters of the primary winding *L_P_* were measured and modeled from the resolver used in this study. From Figure 3, the phase lag *θ_P_* occurs between the excitation signal *v_in_* and the signal *v_RC_* due to the behavior of the resolver. Therefore, the resolver behavior can be approximated by a single-pole expression as

(14) $$\frac{v_{RC}\left(s\right)}{v_{in}\left(s\right)}=\frac{(1-\alpha \Delta T)k_{C}k_{R}}{\left(T_{R}s+1\right)}$$

for

$$T_{R}=\frac{\tan\theta_{P}}{\omega_{ex}}$$

where *T_R_* is the time constant of the resolver. From Equation (14), the circuit in Figure 2b affected by temperature can be represented by the block diagram shown in Figure 2c, where α*_R_* = α*k_R_*.

## 3. Temperature-Compensation Technique

**Figure 4:**
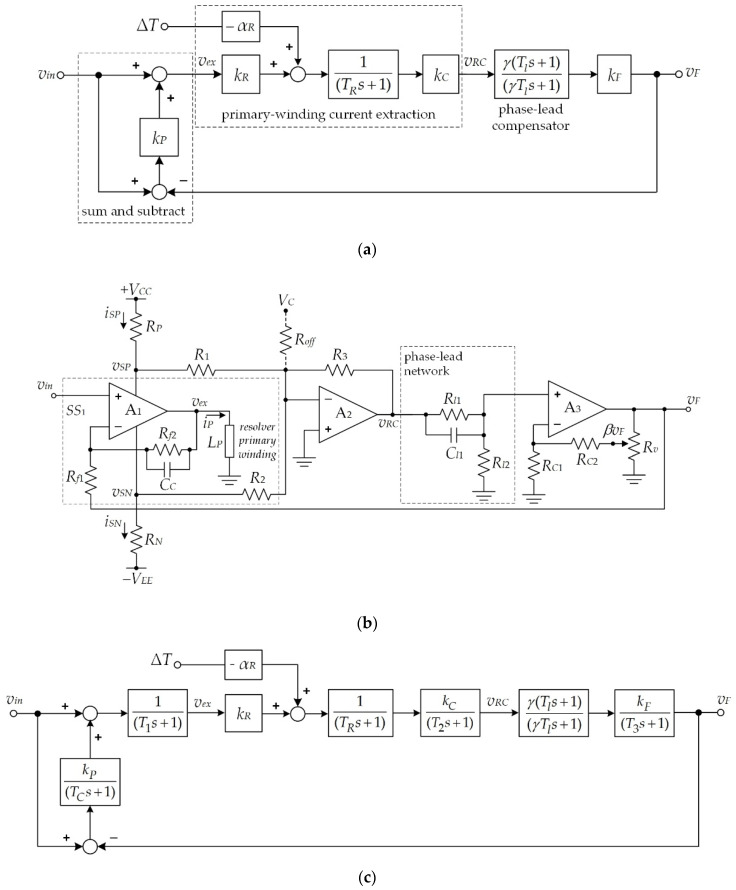
(**a**) Principle of the temperature-compensation technique; (**b**) the proposed circuit; (**c**) block diagram of the proposed circuit.

A simple closed-loop configuration to compensate for the temperature effect is shown in Figure 4a, where the subtract-and-sum topology is depicted within the dashed-line frame. The phase-lead compensator in Figure 4a is required to compensate for the phase lag *θ_P_* of the resolver’s behavior. Therefore, the time constant *T_l_* of the phase-lead network is set to equal the time constant *T_R_* for the in-phase condition between the signals *v_F_* and *v_in_*. From Figure 4a, the closed loop transfer function can be stated as

(15) $$v_{F}(s)=\frac{\left(1+k_{P}\right)\gamma k_{C}k_{F}k_{R}}{\left(1+\gamma k_{C}k_{F}k_{P}k_{R}\right)\left(1+\frac{\gamma T_{l}s}{\left(1+\gamma k_{C}k_{F}k_{P}k_{R}\right)}\right)}v_{in}(s)-\frac{\gamma \alpha_{R}k_{C}k_{F}k_{R}}{\left(1+\gamma k_{C}k_{F}k_{P}k_{R}\right)\left(1+\frac{\gamma T_{l}s}{\left(1+\gamma k_{C}k_{F}k_{P}k_{R}\right)}\right)}\Delta T$$

where *k_P_* and *k_F_* are the proportional gain and the compensation gain, respectively, and *γ* is the attenuation factor of the phase-lead compensator. Normally, *k_C_* = 1/*k_R_* and *k_F_* = 1/*γ* are assigned. If *k_P_* >> 1 and *γk_C_k_F_k_P_k_R_* >> 1, then Equation (15) can be expressed as

(16) $$v_{F}(s)=\left(v_{in}(s)-\frac{\alpha_{R}}{k_{P}}\Delta T\right)\frac{1}{\left(1+\frac{\gamma T_{l}s}{k_{P}}\right)}$$

From Equation (16), the magnitude of the signal *v_F_* for *v_in_* = *V_P_*sin(*ω_ex_t*) can be approximated as

(17) $$\left|v_{F}(j\omega )\right|=\left(V_{P}-\frac{\alpha_{R}}{k_{P}}\Delta T\right)\left[\frac{1}{\sqrt{\left(1+{\left(\frac{\gamma T_{l}\omega_{ex}}{k_{P}}\right)}^{2}\right)}}\right]$$

From Equation (17), the parameters of *γ* = 0.1, *T_l_* = 67.63 μs, and *ω_ex_* = 18.85 krad/s were achieved from experimental implementation. Thus, the denominator of the terms in the square brackets in Equation (17) can be approximated as 1. It is evident that the temperature effect of the primary winding current *i_P_* on the resolver can be minimized by increasing the gain value *k_P_*. The proposed circuit for the block diagram in Figure 4a is shown in Figure 4b. The block diagram of the sum-and-subtract topology in the dashed-line frame in Figure 4a can be replaced by the dashed-line block *SS*_1_ in Figure 4b to minimize the active device used for the experimental implementation.

The proposed circuit consists of three opamps as active devices. The operation of the proposed circuit can be explained as follows: from Figure 4b, the opamp *A*_1_ and resistors *R_f_*_1_ and *R_f_*_2_ function following the sum-and-subtract scheme to obtain the error signal between the input signal *v_in_* and the feedback signal *v_F_*. The capacitance *C_C_* is employed to avoid unstable sum-and-subtract scheme operation [27]. For a small value of the capacitance *C_C_*, the output signal *v_ex_* of the opamp *A*_1_ can be stated as

(18) $$v_{ex}=v_{in}+\frac{R_{f2}}{R_{f1}}\left(v_{in}-v_{F}\right)=v_{in}+k_{P}\left(v_{in}-v_{F}\right)$$

Notably, the signal *v_ex_* is provided as the excitation signal for the resolver. From Equation (18), the signal *v_ex_* is in sum-and-subtract form, corresponding to the sum-and-subtract topology in Figure 4a. The primary winding current *i_P_* is detected by the resistors *R_P_* and *R_N_* in the form of voltage signals *v_SP_* and *v_SN_*, respectively. Opamp *A*_2_ forms the inverting amplifier to obtain the signal *v_RC_* from the *v_SP_* and *v_SN_* signals. The phase-lead compensator formed by capacitor *C_l_*_1_ and resistors *R_l_*_1_ and *R_l_*_2_ provides the phase lead *θ_L_* to compensate for the phase lag *θ_P_* caused by the behavior of the resolver. Opamp *A*_3_, the resistors *R_C_*_1_ and *R_C_*_2_, and the variable resistor *R_V_* provide the compensation gain *k_F_* for the phase-lead compensator. The relationship between the voltage signals *v_RC_* and *v_F_* in Figure 4b for *R_C_*_1_ = *R_C_*_2_ can be expressed as

(19) $$\frac{v_{F}(s)}{v_{RC}(s)}=\frac{2\left(s+\frac{1}{T_{l}}\right)}{\beta \left(s+\frac{1}{\gamma T_{l}}\right)}=\frac{\gamma k_{F}\left(T_{l}s+1\right)}{\left(\gamma T_{l}s+1\right)}$$

where *γ* = *R_l_*_2_/(*R_l_*_1_ + *R_l_*_2_) is an attenuation factor, *k_F_* = 2/*β* is a compensation factor, and *T_l_* = *R_l_*_1_*C_l_*_1_. The compensation factor *k_F_* is set to 1/*γ* for |*v_F_*(*jω*)| = |*v_RC_*(*jω*)|. Notably, the pole at s = −1/*γT_l_* must be far from the left of the zero at s = −1/*T_l_* to prevent phase error at the excitation frequency. Therefore, the factor *γ* ≤ 0.1 is chosen to assign the location of the pole shifted left from the location of the 0 by at least 1 decade. The phase lead *θ_L_* in Equation (19) can be stated as

(20) $$\theta_{L}=\tan^{-1}\left(\omega_{ex}T_{l}\right)-\tan^{-1}\left(\gamma \omega_{ex}T_{l}\right)$$

If *θ_L_* = |*θ_P_*| is set, then the voltage signal *v_F_* and the input signal *v_in_* are in phase.

## 4. Performance Analysis

In practical implementation, a deviation from the ideal performance is caused by the non-ideal characteristic of the devices used in the proposed circuit. The tolerances of the resistors and non-identical supply currents of opamp *A*_1_, *i_SP_*, and *i_SN_* contribute to the amplitude error and the DC offset voltage in the *v_RC_* and *v_F_* signals. The feedback signal *v_F_*, which includes error due to the non-ideal characteristic of the devices used in the circuit in Figure 4b, can be written as

(21) $$v_{F}=\frac{\gamma 2R_{3}R_{C2}R_{P}}{\beta R_{1}R_{C1}}\left(1+\varepsilon_{1}\right)i_{P}+V_{offset}$$

for

$$\varepsilon_{1}=\delta_{A}+\delta_{R}$$

and

$$V_{offset}=\frac{\gamma 2R_{3}R_{C2}R_{P}\left(\Delta_{B}+\Delta_{S}\right)}{\beta R_{1}R_{C1}}$$

where *δ_A_* and *δ_R_* are the tolerances of the resistors *R*_1_, and *R_P_*, respectively; Δ*_B_* and Δ*_S_* are the different currents of the quiescent currents (*I_B_*_1_ − *I_B_*_2_) and the bias currents of the class-AB output state (*I_S_*_1_ − *I_S_*_2_) of the opamp *A*_1_, respectively; and *V_offset_* is a DC offset voltage. Practically, the resistances in Equation (21) are set to *R_C_*_1_ = *R_C_*_2_, *R_P_* = 500 Ω, *R*_1_ = 10 kΩ, and *R*_3_ = 322.7 kΩ, which were chosen for the tolerance of 0.1% or *δ_A_* = *δ_R_* = 0.001. From Equation (21), the error *ε*_1_ can be calculated as 2 × 10^−3^. The effect of the error *ε*_1_ can be minimized by tuning the compensation factor *k_F_* = 2/*β*. The different currents Δ*_B_* and Δ*_S_* are measured from opamp *A*_1_ as about 3.8 and 2.1 μA, respectively. Therefore, the offset voltage *V_offset_* can be calculated as 95.2 mV for *k_F_* = 1/*γ*. The offset voltage *V_offset_* can be cancelled by adjusting the voltage *V_C_* in Figure 4b to an appropriate value. The tolerance of the resistors in the proposed circuit also induces harmonic distortion in the feedback signal *v_F_* for the amplitude of the primary winding current *i_P_* varying in the range of ±2*I_S_*. The total harmonic distortion (THD) as a percentage of the feedback signal *v_F_* for −2*I_S_* ≤ *i_P_* ≤ 2*I_S_* can be expressed as

(22) $$\mathrm{THD}=\frac{\left(\delta_{A}+\delta_{R}+\delta_{S}\right)i_{P}}{16\left(1+\frac{\delta_{A}+\delta_{R}}{2}\right)I_{S}}\times 100\%$$

where *δ_S_* = Δ*_S_*/*I_S_*. From Equation (22), the maximum percentage of THD occurs when the amplitude of the primary winding current |*i_P_*| is equal to 2*I_S_*. If the class-AB bias current *I_S_* of opamp *A*_1_ is 1.302 mA, then the THD percentage will be about 0.09%. Notably, the effect of THD can be prevented. The second factor is the unstable operation of the proposed circuit caused by intermodulation distortion (IMD) and phase shift due to the behavior of the opamps. IMD occurs on the *v_ex_* signal due to the intrinsic pole of the opamp *A*_1_. To avoid IMD, a capacitance *C_C_* is connected in parallel to the resistance *R_f_*_2_ to generate the dominant pole for the sum-and-subtract *SS*_1_. The dynamic behavior of the opamps *A*_1_, *A*_2_, and *A*_3_, caused by the gain bandwidth product (*GBP*) and the gains *k_P_* and *k_F_*, introduces a phase shift in the feedback signal *v_F_*. Figure 4c shows a block diagram of the proposed circuit where the dynamic behaviors of the opamps are depicted. The time constants *T*_1_, *T*_2_, and *T*_3_ of the opamps *A*_1_, *A*_2_, and *A*_3_, respectively, depend on the amplification gains and *GBP*s of each opamp. From Figure 4b, the time constant *T_C_* = *R_f_*_2_*C_C_* is obtained. In addition, the corner frequency *ω_C_* of the term (*T_C_s* + 1)^−1^ should be assigned in the range of 10*ω_ex_* ≤ *ω_C_* ≤ 0.1*GBP*_1_ to avoid the unstable operation of the proposed scheme. Practically, the time constant *T_C_* = (0.1*GBP*_1_)^−1^ is assigned. The time constant *T_l_* = *T_R_* is provided to compensate for the phase lag of the primary winding in the resolver structure. The time constants *T*_1_, *T*_2_, and *T*_3_ can be stated as

(23) $$T_{1}=\frac{\left(1+k_{P}\right)}{GBP_{1}}$$

(24) $$T_{2}=\frac{\left(1+k_{C}\right)}{GBP_{2}}$$

and

(25) $$T_{3}=\frac{\left(1+k_{F}\right)}{GBP_{3}}$$

where *GBP_i_* is the gain bandwidth product of opamp *A_i_* used in this paper. From Equations (23)–(25), the time constants *T*_1_, *T*_2_*,* and *T*_3_ are proportional to the value of the gains *k_P_*, *k_C_*, and *k_F_*, respectively. Notably, a high value of *k_P_* is used to minimize the temperature effect. In addition, high values of *k_P_* and *k_F_* cause the proposed scheme to operate unstably. The phase shift caused by the pole at s = −1/*T*_1_ should approach 0° at the excitation frequency *ω_ex_* to avoid unstable operation. Therefore, the time constant *T*_1_ is assigned to be less than at least 1 decade for the period of the excitation frequency *ω_ex_* or *T*_1_ ≤ 1/10*ω_ex_*. From Equation (19), the pole at s = −1/*γT_l_* of the phase-lead compensator should be placed to the left of the 0 at s = −1/*T_l_* by more than 1 decade by adjusting the factor *γ* to prevent phase error. Therefore, the maximum values of the amplification factors *k_P_* and *k_F_* for maintaining the stable operation of the proposed technique can be expressed as

(26) $$k_{P}=\frac{GBP_{1}}{10\omega_{ex}}-1$$

and

(27) $$k_{F}=\frac{GBP_{3}}{10\omega_{ex}}-1$$

From Equations (26) and (27), the maximum value of *k_P_* and *k_F_* for the stability condition is 132.33 for *ω_ex_* = 18.85 krad/s *GBP*_1_ = *GBP*_2_ = *GBP*_3_ = 25.13 Mrad/s. From Equations (23) and (25), the time constants *T*_1_ and *T*_3_ are calculated as 4.02 and 0.44 μs for *k_P_* = 100 and *k_F_* = 10, respectively. The compensated gain *k_C_* is set to 32.27. Therefore, the time constant *T*_2_ can be determined from Equation (24) as 1.32 μs. From Figure 4b, the resistances *R_f_*_1_ and *R_f_*_2_ are assigned as 2 and 200 kΩ for the proportional gain *k_P_* = 100, respectively. If the time constant *T_C_* = *R_f_*_2_*C_C_* is set to 0.1*GBP*_1_, about 0.4 μs, then the capacitance *C_C_* can be determined as 1.99 pF. The phase shifts *θ*_1_, *θ*_2_, and *θ*_3_ of the poles at s = −1/*T*_1_, s = −1/*T*_2_, and s = −1/*T*_3_, respectively, can be described by

(28) $$\theta_{1}=-\tan^{-1}\left[\frac{\omega_{ex}(1+k_{P})}{GBP_{1}}\right]$$

(29) $$\theta_{2}=-\tan^{-1}\left[\frac{\omega_{ex}(1+k_{C})}{GBP_{2}}\right]$$

and

(30) $$\theta_{3}=-\tan^{-1}\left[\frac{\omega_{ex}(1+k_{F})}{GBP_{3}}\right]$$

For *k_P_* = 100, *k_F_* = 10, and *k_C_* = 32.27, the phases *θ*_1_, *θ*_2_, and *θ3* are −4.33°, −1.43°, and –0.47°, respectively. These phase shifts deteriorate the stability of the proposed technique but can be compensated by increasing the phase *θ_L_* of the phase-lead compensator. Therefore, the time constant *T_l_* of the phase-lead compensator can be written as

(31) $$T_{l}=\frac{\tan\left(\left|\theta_{P}\right|+\left|\theta_{1}\right|+\left|\theta_{2}\right|+\left|\theta_{3}\right|\right)}{\omega_{ex}}$$

For *θ_P_* = −51.89°, the time constant *T_l_* can be determined as 85.3 μs.

In addition, the temperature effect causes deviation of the winding inductance, which is exhibited in the phase shift term *φ_D_* in the primary winding current. The phase shift *φ_D_* can be approximated by the first-order pole at s = −1/*T_D_*, where *T_D_* = (tan *φ_D_*)/*ω_ex_*. The effect of phase shift *φ_D_* in the time constant *T_D_* can be included in Equation (17) as

(32) $$\left|v_{F}(j\omega )\right|=\left(V_{P}-\frac{\alpha_{R}}{k_{P}}\Delta T\right)\left[\frac{1}{\sqrt{\left(1+{\left(\frac{\left(\gamma T_{l}+T_{D}\right)\omega_{ex}}{k_{P}}\right)}^{2}\right)}}\right]$$

Practically, the time constant *T_D_* is much less than the time constant *γT_l_* and can be omitted. From Equation (32), the denominator of the term in the square brackets can be approximated as 1, corresponding to Equation (17). Therefore, the performance of the proposed scheme is unaffected by the phase shift *φ_D_*.

## 5. Experimental Results

The proposed technique was implemented using commercially available devices. The opamps used in this study were LF351 for opamp *A*_1_ and LF353, comprising two opamps in the same package for opamps *A*_2_ and *A*_3_, where LF351 and LF353 provide a *GBP* of 25.13 Mrad/s. The measured average values of the currents *I_B_*, *I_S_*, Δ*_B_*, and Δ*_S_* for opamp *A*_1_ were 2.39 mA, 1.3 mA, 3.8 μA, and 2.1 μA, respectively. The passive devices were chosen as *R*_1_ = *R*_2_ = 10 kΩ, *R_C_*_1_ = *R_C_*_2_ = 30 kΩ, *R_f_*_2_ = 200 kΩ, *R_f_*_1_ = 2 kΩ, *R_P_* = *R_N_* = 500 Ω, *C_C_* = 2 pF, and a variable resistor *R_v_* = 1 kΩ. All resistors used in the proposed circuit were selected for having a tolerance of 0.1%. The power supply was set to ±12 V. The resolver used to demonstrate the performance of the proposed technique was a SANYO 101-4100 with a transformation factor *k_RS_* of 0.37. The excitation signal was assigned as a 3 kHz sinusoidal wave with 2 V peak amplitude. The load resistors *R_L_*_1_ and *R_L_*_2_ for the resolver were assigned as 100 kΩ. The impedance of the resolver including the impedance of the secondary side referred to the primary side was measured from the primary winding using a HIOKI 3532-50 LCR meter as 16.135 kΩ at room temperature (25 °C). Therefore, the conversion factor *k_R_* was determined as 61.977 × 10^−6^ A/V. From Equation (13), the factor *k_C_* = 1/*k_R_* = 16.135 × 10^3^ V/A was set. Therefore, the resistor *R*_3_ of Equation (13) was determined as 322.7 kΩ for *R_N_* = *R_P_* = 500 Ω and *R*_1_ and *R*_2_ = 10 kΩ. The variable resistor was used for the resistor *R*_3_ to achieve 322.7 kΩ. The circuit in Figure 2b was used to determine the dynamic behavior of the resolver.

**Figure 5:**
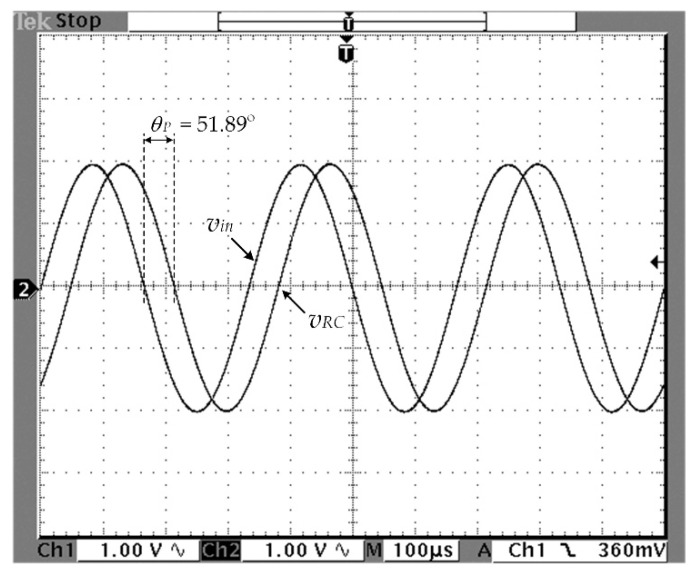
Measured result of signals *v_RC_* and *v_in_*.

**Figure 6:**
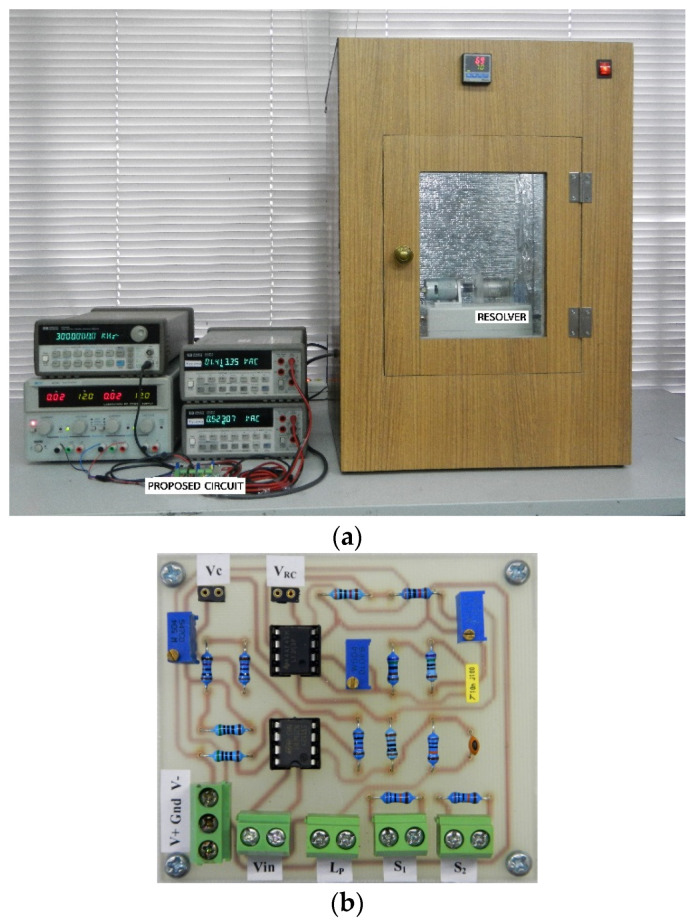
(**a**) Experimental setup; (**b**) prototype of the proposed circuit.

Figure 5 shows the measured result of the phase shift between the signal *v_in_* and the primary winding current represented by the signal *v_RC_*. From Figure 5, the phase lag *θ_P_* between signal *v_in_* and signal *v_RC_* was measured as 51.98° at a room temperature of 25 °C. Therefore, the primary winding impedance including the impedance of the secondary side referred from the primary side can be provided in polar form as 16.135 kΩ ∠ −51.98°. The time constant *T_R_* representing the dynamic behavior of the circuit in Figure 2b was calculated from Equation (14) as 67.63 μs. From Figure 4b, the time constant *T_l_* = *T_R_* was assigned to compensate for the phase lag due to the total impedance of the resolver. The attenuation factor *γ* = 0.1 was chosen in this experiment. Subsequently, *γT_l_* = 676 μs was obtained. The resistances *R_l_*_1_ and *R_l_*_2_ were determined as 6.76 kΩ and 676 Ω, respectively, for *C_l_*_1_ = 0.01 μF. In this experiment, the resistances *R_l_*_1_ and *R_l_*_2_ were chosen as 6.8 k and 680 Ω, respectively. Therefore, the attenuation factor *γ* was recalculated as 0.09. The feedback gain *k_F_* was determined as 11.11 by adjusting the variable resistor *R_v_*. The operation environment of the resolver in this experiment was a chamber capable of varying the temperature from 25 to 70 °C. The shaft angle *θ_S_* of the resolver was set to 45° to obtain equal amplitudes of the secondary winding signals *v_S_*_1_ and *v_S_*_2_. The experimental setup and the prototype of the proposed circuit in Figure 4b are shown in Figure 6a and 6b, respectively.

**Figure 7:**
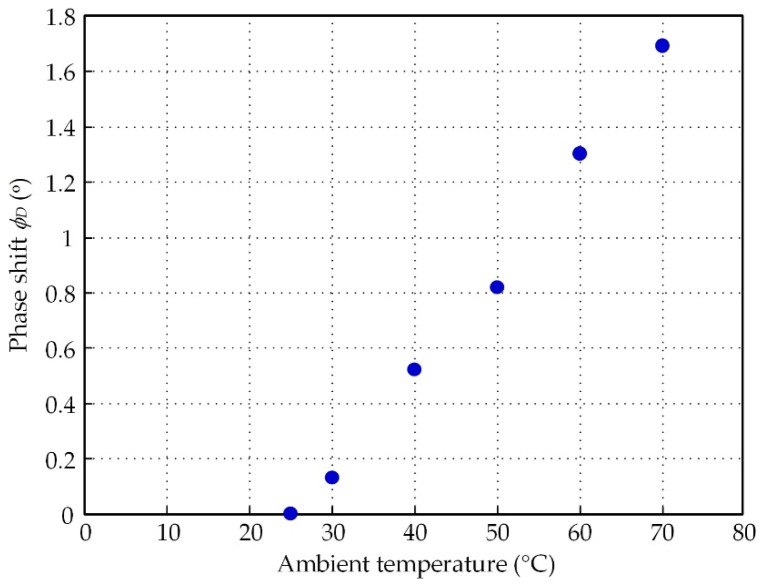
Measured result of phase shift *φ_D_*.

If phase *θ_P_* of the primary winding current at 25 °C is set as a phase reference, the phase shift *φ_D_* of the primary winding current shift from the phase reference *θ_P_* can be seen in Figure 7 for the variations in the ambient temperature from 25 to 70 °C. From Figure 7, the maximum value of the phase shift *φ_D_* at 70 °C is about 1.78°, which corresponds to the time constant *T_D_* = 1.649 μs. The time constant *γT_l_* = 676 μs is much larger than the time constant *T_D_*. Notably, the effect of the phase shift *φ_D_* on the circuit performance is insignificant and can be neglected.

**Figure 8:**
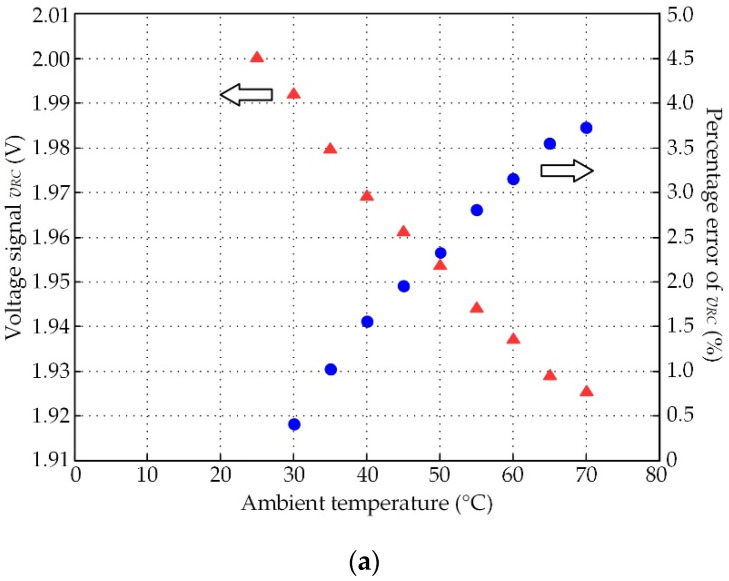

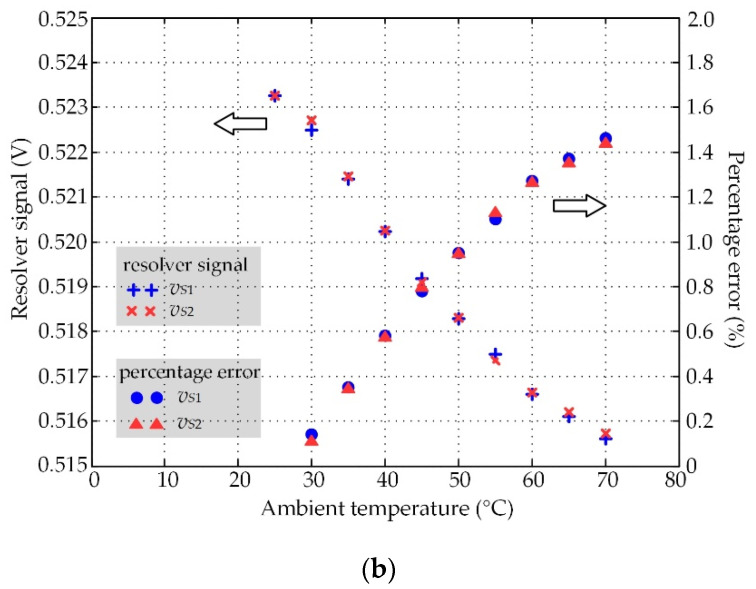
Measured results and percentage errors of resolver signals; (**a**) voltage signal *v_RC_*; (**b**) resolver signals *v_S_*_1_ and *v_S_*_2_.

The phase shift *φ_D_* can be inferred from the change in the resolver inductance. From Figure 7, the variation in the ambient temperature had little influence on the winding inductance of the resolver, in agreement with a recently reported approach [25]. The measured result of the primary winding current in Figure 2a, represented by the signal *v_RC_* versus the variation in the ambient temperature, is shown in Figure 8a, which was used to determine the temperature coefficient *α* of the resolver. The temperature coefficient *α* of the resolver in the term of *α_R_* was approximated from the signal *v_RC_* as −1.71 mV/°C for a 2 V peak amplitude of the excitation signal. The temperature effect on the secondary winding signals *v_S_*_1_ and *v_S_*_2_ was also measured as shown in Figure 8b. The percentage error for this experimental result can be described as

(33) $$percentage\;error=\frac{\left|expected\;value-measured\;value\right|}{expected\;value}\times 100\%$$

As shown in Figure 8a,b, the magnitude of signals *v_RC_*, *v_S_*_1_, and *v_S_*_2_ decreased with increasing ambient temperature. The percentage errors at 70 °C of the signals *v_RC_*, *v_S_*_1_, and *v_S_*_2_ were 3.728%, 1.463%, and 1.44%, respectively. These errors are too high for precision control systems. Therefore, the resolvers require temperature compensation. From Figure 8b, the effect of temperature variation on the resolver signal *v_S_*_2_ was the same as on the resolver signal *v_S_*_1_. Therefore, only the resolver signal *v_S_*_1_ was used to demonstrate the performance of the proposed technique.

**Figure 9:**
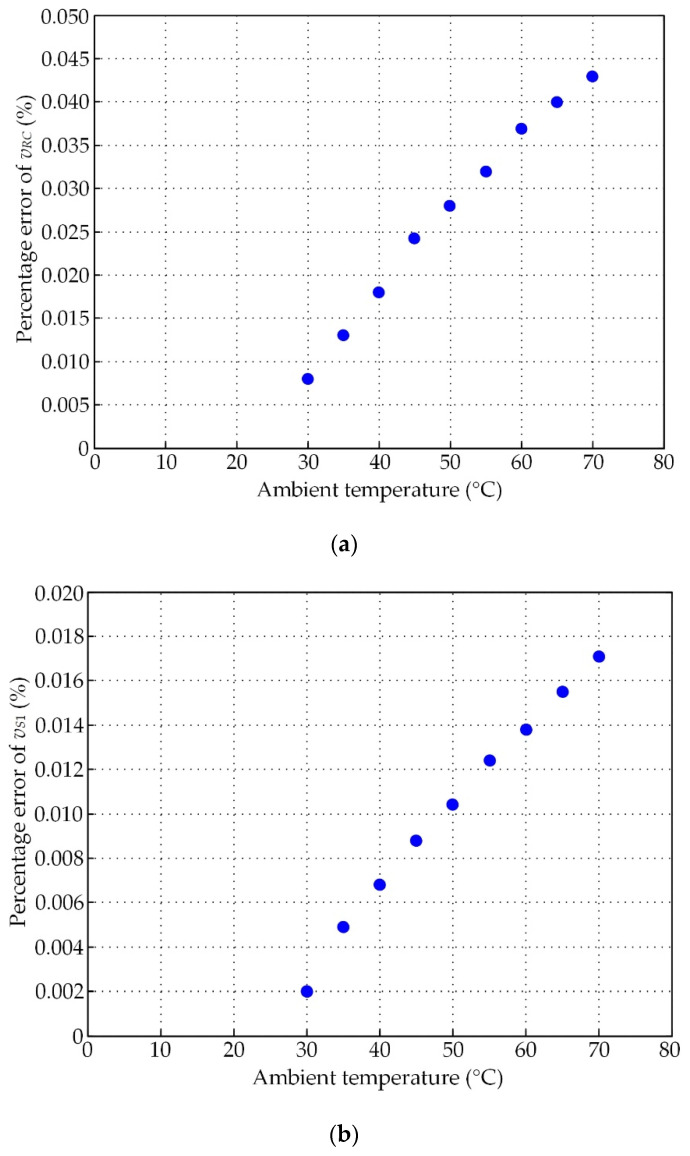

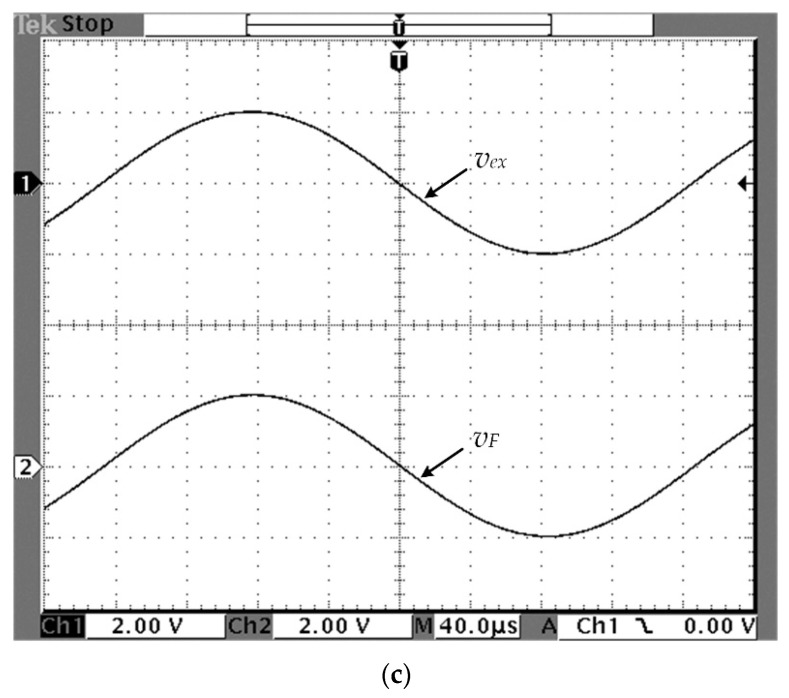
Percentage errors of resolver signals with temperature compensation using the proposed technique: (**a**) voltage signal *v_RC_*; (**b**) resolver signal *v_S_*_1_; (**c**) waveform of signals *v_ex_* and *v_F_*.

The prototype board in Figure 6b was connected to the resolver for the investigation of the circuit’s performance, where the operation environment of the resolver was the same as in the above-described experiment. The experimental results for both signals *v_RC_* and *v_S_*_1_ in terms of percentage error are provided in Figure 9a,b, respectively. Figure 9a,b show that the errors of the signal *v_RC_* and the resolver signals were significantly reduced. Figure 9c depicts the waveform of the excitation signal *v_ex_* and the signal *v_F_* for an ambient temperature of 70 °C. The signals *v_ex_* and *v_F_* were in phase. From Equation (32), the time constant *T_D_* was weighted by the proportional gain *k_P_*. Therefore, the performance of the proposed circuit was independent of the time constant *T_D_* or the phase shift *φ_D_*. As shown in Figure 8a and Figure 9a, the percentage error of the primary winding current represented by the signal *v_RC_* could be minimized from 3.728% to 0.043% at 70 °C. Additionally, the percentage errors of the resolver signal *v_S_*_1_ in Figure 8b and Figure 9b could be reduced from 1.463% to 0.017%. Obviously, the accuracy of the resolver was improved more than 100-fold using the proposed technique. These experimental results confirm that the proposed circuit provides high-quality performance and offers advantages due to a small and simple circuit configuration. In addition, the proposed circuit can be placed between the resolver and a commercial signal conditioner without disturbing the operation of the system.

## Data Availability

Not applicable.
